# Unifying biophysical consciousness theories with MaxCon: maximizing configurations of brain connectivity

**DOI:** 10.3389/fnsys.2024.1426986

**Published:** 2024-07-29

**Authors:** Jose Luis Perez Velazquez, Diego Martin Mateos, Ramon Guevara, Richard Wennberg

**Affiliations:** ^1^The Ronin Institute, Montclair, NJ, United States; ^2^Institute for Globally Distributed Open Research and Education, Gothenburg, Sweden; ^3^Achucarro Basque Centre for Neuroscience, Leioa, Spain; ^4^Consejo Nacional de Investigaciones Científicas y Técnicas, Santa Fé, Argentina; ^5^Department of Physics and Astronomy, Department of Developmental Psychology and Socialization, University of Padua, Padova, Italy; ^6^University Health Network, University of Toronto, Toronto, ON, Canada

**Keywords:** cognition, consciousness, macrostate, metastability, microstate, network connectivity, neural dynamics, neuroscience

## Abstract

There is such a vast proliferation of scientific theories of consciousness that it is worrying some scholars. There are even competitions to test different theories, and the results are inconclusive. Consciousness research, far from converging toward a unifying framework, is becoming more discordant than ever, especially with respect to theoretical elements that do not have a clear neurobiological basis. Rather than dueling theories, an integration across theories is needed to facilitate a comprehensive view on consciousness and on how normal nervous system dynamics can develop into pathological states. In dealing with what is considered an extremely complex matter, we try to adopt a perspective from which the subject appears in relative simplicity. Grounded in experimental and theoretical observations, we advance an encompassing biophysical theory, MaxCon, which incorporates aspects of several of the main existing neuroscientific consciousness theories, finding convergence points in an attempt to simplify and to understand how cellular collective activity is organized to fulfill the dynamic requirements of the diverse theories our proposal comprises. Moreover, a computable index indicating consciousness level is presented. Derived from the level of description of the interactions among cell networks, our proposal highlights the association of consciousness with maximization of the number of configurations of neural network connections ―constrained by neuroanatomy, biophysics and the environment― that is common to all consciousness theories.

## 1 Introduction –a flood of theories and the need for unification

Whereas some decades ago few respected experimental scientists would consider the investigation of consciousness a worthy scientific pursuit, recent years have seen a surge in the study of consciousness, with natural implications for neuroscience, philosophy, neurology, psychiatry and psychology. As a result of this surge, we are now faced with a surfeit of consciousness theories (Rosenthal, 2020; Seth and Bayne, 2022; Stefanelli, 2023). Some may perceive the abundance of theories as a healthy sign of thinking about consciousness, however, “growth is not always benign […] Cancer is a good example,” as said by philosopher Robert Chis-Ciure in a talk on consciousness theories (Horgan, 2023). While each theory has specific claims and mechanistic details, many of them share a common essence, at least at the biophysical level. Among what are considered to be the four principal types of contemporary scientific theories of consciousness (Seth and Bayne, 2022), a dispassionate reading suggests that the main differences lie in the elements of each theory that do not have a clear neurobiological basis. Specifically, “meta-representation” in the case of higher-order theories (HOTs), a physical “workspace” in the case of global workspace theories (GWTs), “Φ (phi)” in the case of integrated information theory (IIT), and a “top-down predictor” in the case of re-entry and predictive processing theories. One could easily substitute a non-material “mind” for any of these theoretical elements and the predictions of these theories would be unchanged.

In the hope of advancing the scientific study of the biophysical aspects of consciousness, we would argue that what is needed is not more theories, but rather an integration across existing theories to build a comprehensive neurobiological view on consciousness and its related human function, cognition. An integrated theory would also facilitate an understanding of how normal neural dynamics might develop into pathological states, thereby having practical value as well.

In this article, we propose an encompassing theory –derived from a conceptual framework– that incorporates aspects of other current theories, which, as mentioned above, at their essential biophysical level are not really dissimilar. We will call our theory MaxCon for short (for *Max*imizing *Con*figurations of neural connections), finding convergence points in other theories that are often obscured in semantic or technical differences. We think this is needed in our current times when even competitions between consciousness theories seem to be in fashion. Criticisms about a lack of convergence among theories of consciousness have already been voiced (Del Pin et al., 2021); moreover, recent publications have emphasized the need for synthesis in that “theories should not be viewed as rivals, but as partial perspectives on a deeper mechanism” (Graziano et al., 2020), and some have proposed creating minimal unifying models (Wiese, 2020). Along these lines, we will propose a unifying biophysical theory that describes necessary and sufficient conditions for consciousness and, relatedly, cognition.

The nature of consciousness –and the related characterization of the workings of the brain– is considered to be an extremely complex problem. Therefore, one needs to find a perspective from which the subject appears in relative simplicity, a practice recommended by one of the founding fathers of statistical mechanics, J. W. Gibbs, who wrote in a letter to the American Academy of Arts and Sciences in 1881: “One of the principal objects of theoretical research […] is to find the point of view from which the subject appears in its greatest simplicity.” We must consider that relatively simple laws may emerge out of extremely complex processes, for even lawless phenomena have regularities, as pointed out by Gnedenko and Kolmogorov (1954): “All epistemologic value of the theory of probability is based on this: that large-scale random phenomena, in their collective action, create strict non-random regularity.” The simple perspective we take here, as explained below, is that of the interactions among brain cell networks at the meso/macroscale.

Observations have accumulated to indicate that a foundation of consciousness consists in the widely distributed propagation of cellular activity in the nervous system, specifically the brain. This is probably the only aspect on which everybody would currently agree. In addition, during normal wakefulness, this distributed neural activity should be variable enough to allow the organism to experience the irregularity of the environment. The experimental evidence for disrupted neural connectivity (see next section for a comment on “connectivity”) in unconscious states such as sleep, anesthesia and other disorders of consciousness is considerable, these being characterized by a constrained repertoire of functional brain states (Mashour and Hudetz, 2018) along with decreased complexity of these states (Sarasso et al., 2021).

Therefore, considering the dynamic nature of consciousness, and that of cognition (Craik, 2020), both derived –in neurobiological terms– from the configurations of functional neural network connections as the fundamental building blocks of nervous system function, we propose to advance a wide-ranging biophysical theory of consciousness (and cognition) through the perspective of an *integrated dynamic process capable of achieving a large number of configurations of functional connections*. The global underlying idea is that the number of functional network configurations represents a simple measure of the computational flexibility of a nervous system. We will bring to the fore commonalities among several of the main existing theories and, along the lines of a global principle of the organization of neural dynamics (Perez Velazquez et al., 2019, 2020, 2023), we will explore how these different aspects of the theories fit into the one we propose, backed up by experimental observations. For this purpose, we will examine how neuronal cellular collective activity organizes to fulfill the dynamic requirements of the diverse consciousness theories that are encompassed by our own. Unlike some other current theories, we do not posit a localized brain area where consciousness takes place; rather, making consciousness a property of the whole brain gives us the chance to postulate specific ways in which different types of consciousness may exist, and possibly to better understand the nature of cognition, behavior, and subjective awareness. Finally, we will discuss the practical use of this knowledge to potentially improve health in patients with alterations ofconsciousness.

## 2 Some groundwork

For the sake of precision, let us initially clarify some essential terms and concepts. Obviously, consciousness will be our main concern and therefore we point out that consciousness is an arbitrary concept developed from efforts to encapsulate a constellation of phenomena. Considering that the notion of consciousness has been around for centuries, the fact that there is no strict definition widely accepted by a majority of scientists and philosophers is already telling us something, specifically, that perhaps there is no definitive definition. Indeed, consciousness means different things to different people (Lenharo, 2024). At the same time, everybody accepts the fact that consciousness science is a vastly interdisciplinary theme involving many disciplines ranging from neuroscience and psychology to computer science and mathematics, thus finding a strict, succinct definition that could be assented by all the practitioners in these different fields may be next to impossible. If finding a precise and concise one-sentence definition is barely feasible, then defining it by enumerating its features –starting with the primordial sensing and responding to stimuli and continuing with attention, memory, choice-making and perhaps the summit of this (arbitrary) hierarchy, the experience of agency and self-awareness– is a possible solution; among other things, this makes reasonable the idea that consciousness can be scientifically studied (Perez Velazquez, 2020; chapter 7 in Perez Velazquez and Nenadovic, 2021), because many of the properties just mentioned can be empirically studied and even quantified, such as responsiveness (with the coma scales) or attention. In this regard, other authors seem to think this to be a reasonable and practical manner in which to attempt to define consciousness, as exemplified in the words of Anil Seth: “Biologists have simply gotten on with the business of explaining the various properties of living systems in terms of underlying mechanisms: metabolism, homeostasis, reproduction and so on. An important lesson here is that life is not ‘one thing’ –rather, it has many potentially separable aspects” (Seth, 2016). Similarly, as Paco Calvo has expressed while discussing plant cognition: “consciousness […] is to be understood in a wider context […] as various aspects of mentality, including subjective experience, awareness, inner representation, central coordination, goal-directedness, memory, self-awareness” (Calvo, 2017). Others have cast doubt on considering consciousness as a single informationally unified cognitive capacity (Montemayor, 2021).

All in all, we consider consciousness and cognition not as all-or-none phenomena; instead, we advise taking an evolutionary perspective because in so doing some problematic issues inherent in this topic can find solution (Feinberg and Mallatt, 2018). In our text, and having in mind the aim to simplify matters, we will consider consciousness as conscious awareness because talking about consciousness is talking about the capacity to experience one’s environment and internal states; after all, being conscious entails being conscious of something, that is, to be aware of something. In fact, in dictionary definitions of consciousness (e.g., Webster’s Third New International) the word ‘awareness’ always appears. We note there is some debate about the overlap between consciousness and awareness, precisely due to the aforementioned fact that consciousness means different things to different people. Sometimes we write “*consciousness/cognition*” because we consider that these two terms are so much intertwined –two sides of the same coin– that to separate them would be to dichotomize in the extreme, and creating dichotomies is a constant ailment in neuroscience (Kelso and Engstrøm, 2006).

Another concept we will use is that of brain state, as we will be referring to micro and macrostates of neural activity. We will also use some concepts from statistical mechanics, but in reality these are not needed to understand the basic message of our theory. As with theories of consciousness, there is a wide variety of concepts of brain state; for an attempt to reach a comprehensive conceptualization of brain states see Greene et al. (2023). Again with the aim to simplify, we will consider a *brain state* any configuration of cellular activity, basically any transient clustering of cells according to a similar activity profile (Sadaghiani et al., 2010). This is reasonable considering that configurations of neural network connections are the basic building blocks of nervous system function.

Since we will adopt a perspective based on the interactions between cells or networks of cells –what can be considered a connectionist approach–, the term connectivity will appear throughout the text. For the sake of simplicity we will refer to *connected networks* those that have correlated activity. And by “*network*” *we denote either the group of cells that generate the collective neurophysiological signal that may be detected by a sensor, or a particular brain region* (or equivalently a source in brain tissue that has been determined by some sort of source localization process).

## 3 The fundamental perspective

Before beginning to discuss several theories and frameworks of consciousness we should highlight the aspects of nervous system activity we will be focusing on. Neurophysiological recordings of brain activity demonstrate fluctuating patterns of cellular interactions (the waveforms of any electrophysiological recording reflect, predominantly, synaptic interactions among neurons), variability that allows for a wide range of states, or configurations of connections of distributed networks exchanging information, which support the flexibility needed to process sensory inputs and execute motor actions. Hence, the viewpoint that we use to discuss the diverse theories and support our proposal is what we think is one of the simplest: the meso/macroscale level of description (referring to measures of the collective activity in large cell populations) will be the focus in terms of the dynamics of the collective cellular activity of the nervous system (and indeed almost all theories are centered at that scale). This perspective is reasonable given the universal acceptance that the biophysical foundation of consciousness consists in the propagation of cellular activity through the nervous system –this activity being modulated or constrained by inputs from the rest of the body and from the environment. That consciousness relies on large-scale cellular communication is common to several theories as we will see below (Tagliazucchi, 2017), which is something that can also be said about almost all, if not all, living systems, being characterized by the emergence of patterns due to the interplay between short and long-range interactions and correlations among the system’s constituents.

The question is how best to describe the organizing principles of this cellular collective activity that allow the features of consciousness to emerge. What is the optimal organization of brain activity that allows it to adequately process sensory stimuli and enables the organism to adapt to its environment? In this text we will advocate several times the physicist’s approach to understanding natural phenomena; first, to seek laws based on abstraction, which is something needed in neuroscience to make sense of the overabundance of data. Because rather than a description, it is a fundamental understanding at a high level of description that we seek –a high level that emerges from numerous particular lower-level mechanisms– and whereas a description is a good approach to understanding, it is not quite the same thing (Laurent, 2000), and the choice of the level of description will dictate the nature of the understanding.

**Figure 1:**
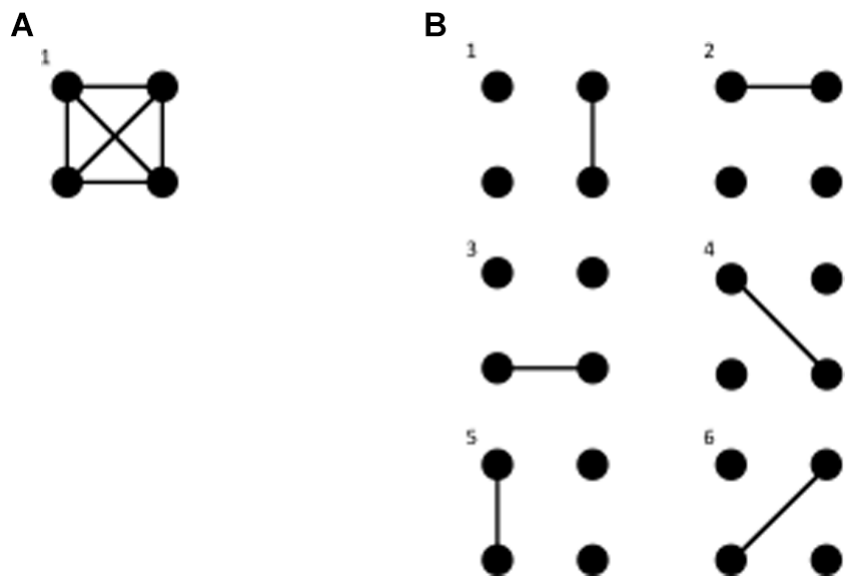
Brain microstates as connectivity configurations. Each dot represents a cell network or a signal recorded in a sensor (or equivalently a source in brain tissue) and the lines a connection between them (e.g., synchronous activity). In **A**, with a complete connection of all networks, there is only one microstate, one possible configuration of connections. In **B**, with connectivity restricted to 2 networks, we find 6 possible microstates.

In the next section several theories will be examined using the framework abovementioned based on interactions at the meso/macroscopic level, but we need to be even more precise. Considering experimental results that will be described below and the fundamental tendency of natural phenomena toward equilibrium, we posit a principle of organization of neural dynamics. Following the classic approach in physics when it comes to understanding collective behaviors of systems composed of a myriad of units, we suggest paying attention to the possible configurations –*microstates*– that the system can adopt. Consequently, the proposal is that an *organizing principle of cell assemblies in nervous systems is a tendency to maximize the number of configurations of connections (microstates) among cell networks*, obviously constrained by the biophysical and structural properties of the constituent cells and by the environment where the nervous system resides; wakeful states should be characterized by the tendency to express the greatest number of possible configurations of interactions between brain networks, whereas unconscious states should have a lower number of configurations. We will see in the next section that this notion lurks behind many existing theories of consciousness. To clarify what we term a microstate, please see Figure 1.

More technical explanation of some of the concepts in sections 2 and 3 and further clarification about our line of thought are presented in the Supplementary material.

## 4 Theories of consciousness: a unifying view

Considering the deliberations of the previous sections, let us explore the possibility of searching for a wide-ranging biophysical theory of consciousness. We will discuss several theories and frameworks which, if their essence is distilled, share common aspects that give us hints as to the principles of neural organization, and how these align with our proposal. We emphasize that the intention is not to review in detail the existing theories, but rather to extract a main essence that may be compatible with our organizing principle aforementioned and with our own theory to be described in the next section. Therefore, *we will focus on common aspects of the theories/frameworks and not on differences* because the latter are many, often subtle and/or non-biological and impossible to address in a paper that is not intended to be a review. Nevertheless, we will mention a few crucial differences with some theories, such as the location of consciousness (section 5.3).

Since in our theory we are taking the neurophysics/neurophysiological perspective, *we will concentrate only on those theories that are expressible in neurobiological terms*, so the enormous variety of philosophical content theories will not be discussed here given that most are not too much anchored in empirical observations. Again, we will follow a practice in physics: when theories are not compatible but overlap to some extent, the ideas may be refined and lead to a single better theory. In the next section we will propose our framework and then we will examine whether it is backed up by experimental observations. In that we will consider consciousness as conscious awareness, we will focus the appraisal of theories on the question of what makes a representation conscious, and not so much on accounts of phenomenal differences between distinct experiences.

One framework that perhaps most clearly suggests the fundamental importance of the variability in neural activity resulting in large numbers of connectivity configurations is the metastability conceptual framework. Metastability in neuroscience refers to a type of dynamics characterized by patterns that reappear either in repeatable sequences or in apparent randomness (Kelso, 1995; Hancock et al., 2023). This framework has as an underlying notion the need for a substantial number of configurations of cell connections conferring on brain areas more variability to establish different connectivity patterns for proper sensorimotor transformations. As a corollary, when the activity is too stable and becomes trapped in one pattern for a relatively long time, as occurs during epileptic seizures, status epilepticus, coma or during slow wave sleep, unconsciousness ensues. Hence, our proposed high variability of connectivity configurations, thus making brain states quasi-stationary states, can be considered a mark of metastable regimes.

Higher-Order Theories (HOTs) have as a central claim that mental states are conscious in virtue of meta-representations: higher-order representations that represent something about other, first-order representations, which represent things about the world like auditory or visual signals (Brown et al., 2019). Lower-order representations in different brain regions are envisioned as being conscious when “targeted” by specific meta-representations hypothetically localized in prefrontal cortical areas. The proposal of HOTs implies widely distributed, long range cellular connectivity coordinating lower-order and higher-order representations, suggesting that relatively high variability in the establishment of connections among brain areas is a necessary, and perhaps sufficient, aspect of consciousness.

The Global Workspace Theory (GWT) essentially claims that widespread distribution of information (cellular activity) leads to conscious awareness. The fundamental premise is that information processed in various isolated and unconscious cognitive modules within the brain becomes conscious when it is “broadcast” within interconnected frontal–parietal neural networks, allowing access to many other cognitive systems, with the contents of consciousness selected and amplified (“ignited”) by attention to specific signals in the workspace. The selective attention and other related cognitive effects on the workspace, e.g., working memory, are putatively localized to prefrontal cortical areas (Baars, 1993). And how do we build a global workspace that can access that widespread distribution of information (which is cellular activity, in the final analysis)? Increasing the number of possible configurations of neural connections is one immediate solution. So once again the underlying notion to broadly broadcast activity to many different cell networks throughout the brain demands sufficient variability, flexibility, in neural connectivity.

Contrary to the GWT and to HOTs, the Integrated Information Theory (IIT) associates consciousness primarily with a posterior cortical temporal–parietal-occipital zone. According to this theory, consciousness is integrated information (Tononi, 2004). The important point for our purposes is that according to the theory, consciousness increases in proportion to the system’s repertoire of states, requiring an assemblage of elements within a system that have “cause-effect power” upon one another, which implies the need for re-entrant architecture consisting of feedback loops; this basically constitutes integrated information, which in the IIT is associated with the theoretic quantity Φ (proposed as a measure of level of consciousness). Therefore, distilling the essence again, we find as an underlying notion of this framework the need for a substantial number of microstates, or configurations of cell connections, in order to increase the integrated information. We find then the same notion as that found by inspecting the essence of the HOTs and GWT frameworks/theories (Table 1).

**Table 1:** Principal types of neuroscientific theories of consciousness.

Theory type (reference examples; see also Seth and Bayne, 2022 for review)	Explanatory postulates	Putative location of consciousness in the brain	Requirement for large number of configurations of cellular connectivity
Higher-Order (Brown et al., 2019)	Meta-representation	Prefrontal cortex	Yes
Global Workspace (Baars, 1993)	Broadcast, ignition Workspace	Frontal–parietal networks Prefrontal cortex	Yes
Integrated Information (Tononi, 2004)	Cause-effect structure Irreducibility (Φ)	Temporal–parietal-occipital cortex	Yes
Re-entry/Predictive Processing (Edelman and Gally, 2013)	Top-down signaling Predictive inference	Diffuse, non-localized Thalamo-cortical networks	Yes

The notion of re-entry has appeared above while discussing the IIT. There are several re-entry theories. The Re-entrant Processing Theory proposes that re-entrance of activity in neural circuits serves a role of integration and can happen across the global brain hierarchy (Edelman and Gally, 2013). Re-entry goes under other names like reverberant, recurrent, reafferent, or feedback processing. These theories are motivated by neurophysiological evidence revealing the importance of top-down signaling for conscious awareness (Lamme, 2006), and also by the well-known recurrent structural (neuroanatomical) features present at almost all levels in the nervous system, from central pattern generators to the cerebral cortex. The principal claim of re-entry theories (including the Predictive Processing Theory) is that conscious mental states are associated with top-down signaling (re-entrant neural activity) or perceptual predictions. Another theory relying on re-entrant activity is the Dynamic Core Theory that postulates re-entrant activity in thalamo-cortical networks giving rise to conscious experience (Edelman et al., 2011) and an integrated dynamic process capable of achieving a vast number of configurations (Tononi and Edelman, 1998). Thus, we find again the common aspect of a “vast number of configurations” of neural activity providing the required variability/flexibility in nervous system dynamics to process sensorimotor transformations (Table 1).

Some other notable theories include the Temporo-spatial Theory of Consciousness (TTC) (Northoff and Huang, 2017) which has two main aspects that suggest, similar to all of the previous frameworks, a fundamental role for fluctuations in neural activity in terms of connectivity. These two aspects are “temporo-spatial expansion” and “temporo-spatial globalization,” the former being a spreading out of early stimulus-induced activity which accounts for phenomenal consciousness and the latter a globalization of late stimulus-induced activity which accounts for the cognitive features of consciousness. The expansion and resulting globalization of activity would most likely require a variable dynamic state space to sustain enough configuration patterns of activity.

The Self-organizing Metarepresentational Account (SOMA) theory, in which consciousness is viewed as something that the brain learns to do, has the brain continuously and unconsciously learning to re-describe its own activity to itself, thus developing systems of metarepresentations (Cleeremans et al., 2020). One would expect that this re-description entails variability in the configurations of neural connections in order to enable the widespread distribution of information processed in different brain regions for those metarepresentations.

The main postulate of the Dynamical Emergence Theory (DET), which is that distinct perceptual states correspond to coarse-grained brain macrostates reflecting an optimal partitioning of the system’s state space (Moyal et al., 2020) is very similar to our proposal –explained in detail in the next section– in that the constitution of brain macrostates made up by a large number of microstates is crucial for proper sensorimotor transformations associated with conscious awareness. We will end this section mentioning that other authors too have advocated for integrating consciousness theories or models, some cited in the Introduction; for instance Graziano et al. (2020) have a different, not so neurophysiologically-based approach but rather a social cognition approach, trying to make sense of the apparent gap between diverse theories. Another integrating theory, Consciousness State Space (Berkovich-Ohana and Glicksohn, 2014), presents a theoretical phenomenological model for consciousness and selfhood relating time, awareness, and emotion within one framework, but again we cannot compare our proposal to theirs due to our purely neurophysically oriented approach. Finally, Kanai et al. (2019) proposed a global model claiming that information generation could serve as a functional basis for consciousness, a model that has been considered somewhat vague by other authors who have proposed that information generation, which by itself does not provide much insight, could serve as a minimal unifying model (MUM) of consciousness. The notion of a MUM, mentioned in the Introduction, represents another attempt at simplifying the scenario in consciousness research. Our theory could even be considered a MUM, in the sense that it “specifies at least one necessary feature of consciousness, characterizes it in a determinable way, and shows that it is entailed by (many) existing theories” (Wiese, 2020): the necessary (or rather, sufficient, as explained in the next section) feature being a large number of connectivity configurations (the computation of this number is shown below). As we have seen in this section that is a common underlying idea of several frameworks.

## 5 MaxCon: maximizing configurations of the brain web

In the previous section we have claimed that the neurophysiological common theme of several current theories is a fundamental role for the fluctuations, or variability, in neural functional connectivity. We now present our own theory based on that common theme and on experimental observations which will be described mainly in the next section. Our intention is to talk about *specific things that have a clear neurophysiological interpretation*, therefore we will try to avoid terms like complexity, information, self-organization and the like, for the reasons discussed in the Supplementary material, although our descriptions can be interpreted in these terms.

Based on the organizing principle of neural dynamics elaborated in section 3 (principles are abstract ideas that help to formulate theories which constrain the formulation of models), namely that cell assemblies in the nervous system tend to maximize fluctuations and variability in their functional connections (constrained by their biophysical and structural properties and by the environment), we propose that a main mechanism governing the emergence of neural dynamic patterns is the fluctuations in configurations of connections among cell networks. Advocating the approach used in the physics of complex systems, we consider the microstates our system can adopt, and posit that the brain macrostates are comprised of microstates –configurations of neural network connections– and that the number of those microstates tends to maximize with conscious awareness. In addition, not only unconscious states but also some pathological states will display a lower number of configurations of neural connectivity, that is, fewer microstates. The larger number of microstates during conscious awareness affords brain areas more variability to establish different connectivity patterns for proper sensorimotor transformations, as a main function of the brain is to deal with the unforeseeable, multidimensional, complex environment. Figure 1 depicts a pictorial explanation of what we call microstates.

In truth, MaxCon is reminiscent of old proposals like Flohr’s rate of dissolution of neural assemblies determining degrees of consciousness (Flohr, 1991). And going far back in time, the zoologist Charles Bonnet, talking about organization, proposed that an organization realizing the most effects (which can be equivalent to our configurations of connections) with a given number of parts (equivalent to the anatomically established connections) is a most perfect one (Bonnet, 1764). Modern science is many times a re-discovery of past notions, aided by new vocabulary and techniques.

Our basic assumption is that a macrostate is described by the total number of configurations of connected brain regions, these being the microstates. More explicitly, that a brain macrostate is described by macroscopic observables depending only on functional connectivity (this is of course a simplification), whereas the microstates are the possible states accessible to the system. This is a standard approach in thermodynamics, here applied to the specific situation of brain functional connectivity. In essence, then, connectivity defines the macrostate, and different macrostates represent different general states of consciousness, such as normal alertness, seizures, or sleep stages. We do not posit that macrostates defined in this manner can be associated with very specific mental states or behaviors such as running or thinking about music, instead, these macrostates differentiate global behaviors: full awareness, unconsciousness during sleep or coma, etc. However, the many microstates present in wakeful states (as will be detailed in the next section on experimental observations) provide the neural mechanisms which are specific to each sensorimotor transformation required to navigate our complex environment in awake, conscious states.

But then one could also maintain that while each configuration determines a behavior, some configurations may be caused by sensory inputs that in turn determine a behavior, which then and due to proprioception cause another sensory input which has the same or a different configuration associated with it. And even more, considering the abundant evidence of pre-configured neural circuits (Betzel et al., 2012; Chu et al., 2012) one could claim that those microstates are manifestations of these preconfigured circuits that may be modified by sensory inputs and then determine behaviors which in turn cause other sensory inputs... And so on. The advice of Gilles Laurent should always be present when thinking about these issues: “Our thinking generally ignores the fact that […] a given neuron is never an end-point or its response an end-product […] Thinking about sensory integration in these active terms (considering ‘responses’ not only as products but also as ongoing transformations toward some other goal) may be helpful […] to understand some brain operations” (Laurent, 2002). Neither our theory nor any other, to our knowledge, can specify what specific neural connectivity pattern determines each particular sensorimotor transformation.

What at the macroscale may seem relatively stable steady states –those brain macrostates associated with different behavioral conditions– at the local, microscopic level, variability, or fluctuations in network connectivity is what is found: changes in the connectivity configurations –the microstates making up a macrostate. When assessed with the appropriate analytical techniques, the fluctuation at short time scales of the configurations of connected networks becomes evident, and this represents the establishment and dissolution of “connections”; these are precisely the microstates we are talking about that make up a macrostate. In other words, in our approach the macroscopically measured observables that constitute a macrostate have an intrinsic variability due to fluctuations in microscopic variables, such as synaptic plasticity and many others, that determine the formation of each microstate. Hence, in a study that examined the short-time scale –microscopic nature so to speak– of configurations of connections using a complexity measure that allowed for the assessment of instantaneous fluctuations in configurations of connected networks (Mateos et al., 2017) it was found that even in moments of (healthy) global unconsciousness, such as slow wave sleep, these fluctuations (in terms of the complexity value) at short time scales were found to be similar to those during wakefulness, perhaps reflecting the subconscious cognitive processing known to take place during sleep –e.g., memory consolidation (Sejnowski and Destexhe, 2000). To be more explicit: the macrostate associated with slow wave sleep has fewer microstates than that associated with wakefulness: the fewer number of configurations of connections in the brain cannot maintain full awareness, the individual is unconscious (sleeps); but at smaller scales the fluctuations in connectivity may be enough to perform unconscious cognitive processes thought to occur during sleep. In short, we find here the global and local perspectives: the microstates (local view) need to fluctuate and hence are unstable/metastable, whereas the global macrostate remains stable. There is no paradox, it all depends on the level of description. It is therefore in this sense that we say that *consciousness is a global property of brain dynamics*: at global, macroscopic and long-term scales, the macrostate associated with conscious awareness is relatively stable (and the one associated with unconscious states is equally relatively stable), but at finer spatio-temporal scales there is a variability that is also found in healthy unconscious states –but not in pathological unconscious states like coma or seizures (experimental details in the following section).

Thus, in our view, the brain reaches a steady state formed by many microstates (configurations of functional connections), and depending on external and internal constraints including neuroanatomical connectivity, the total energy available and the inputs the brain receives –that is, depending on the context–, this macroscopic steady state will have different features. Among these characteristics, the number of possible configurations of connections will differ, more abundant during conscious awareness and fewer during unconscious states. We will see support for this in the next section. It might therefore be said that embodied brains immersed in an environment seem to attempt to reach equilibrium, which is impossible due to the exchange of energy (information) with the surroundings.

What causes those microstates to be constantly fluctuating? The answer is found in response to this other question: what makes a neuron fire? Another neuron (to be precise, in most cases several neurons firing in synchrony are needed to make one neuron discharge a spike potential). This already hints at a fundamental rule in nervous system structure and function, the recurrence-reverberation mentioned in the previous section. This can be captured by the notion of neural closure, because a nervous system can be considered to be a functionally closed, self-sustained system of cellular networks, a closed web of cell network connections where one can reach any neuron/network in the brain starting from any other (Perez Velazquez, 2020). It is not surprising then that it is distributed brain networks rather than circumscribed centers that mediate most of sensorimotor processing. We also venture that as “neuroglial closure” achieves more complexity in terms of numbers of cells and connections, the cognitive powers of an organism become greater.

All these reflections serve as a ground to propose *a necessary and a sufficient condition for consciousness (and cognition)*: the former is rather trivial, cellular activity (energy); the latter is the organized collective activity of those cells (or the energy distribution, in other words the number of ways that energy –that is, cellular activity– is distributed), in particular the tendency to maximize the number of connectivity configurations and patterns. Neurons use electrochemical energy to communicate. By maximizing connectivity configurations (which implies maximizing the number of ways electrochemical energy is distributed), the brain ensures an efficient process of energy distribution, thus playing a crucial role in supporting the dynamic activity necessary for consciousness and cognition.

In physics jargon, this entails the dissipation of energy (Prigogine, 1955; Guevara et al., 2020). Given the importance of energy dissipation in pattern formation (Haken, 1998), then *to generate the rich dynamic activity necessary for consciousness and cognition, energy gradients need to be established and maintained, albeit with enough fluctuations to allow diverse connectivity patterns*. It is therefore plausible that energy is distributed in more pathways in healthy conditions and during conscious awareness. The importance of the organization of energetic processing in the brain has also been proposed by other authors (Pepperell, 2018; Chen and Zhang, 2021).

### 5.1 A computable index

**Figure 2:**
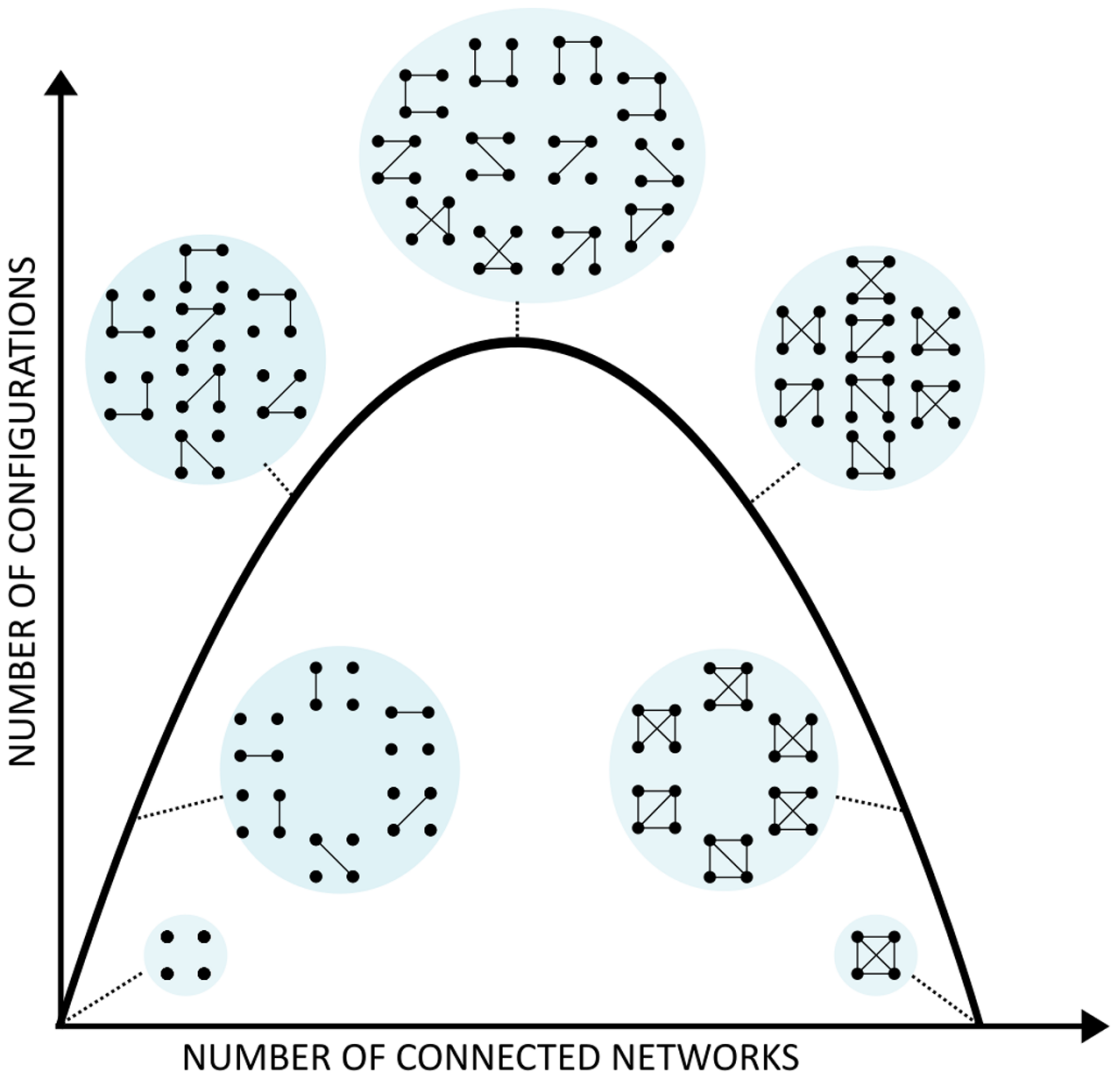
Configurations of connections among cell networks. As in Figure 1, each dot represents a neural network or signal and the lines their connectivity that define microstates, with the macrostate represented as the circle surrounding the microstates. For either no connectivity or all-to-all connections, the number of configurations is low (only 1), as seen at the right and left hand side of the inverted U curve, whereas the maximal number of configurations is found at the top, which is given by a certain number of allowed connections, neither too many (moving toward the right of the curve) nor too few (moving toward the left of the curve).

Figure 2 represents the essence of our theory. The *x* axis represents the number of connected networks (signals), so one can see that there is a maximum in the number of possible connectivity configurations (*y* axis) depending on the connected networks, and this is relative to the number of signals (networks) considered. That is the reason why in conditions of higher synchrony (e.g., during epileptic seizures) with more connected (synchronous) brain areas, the number of configurations is lower than in normal awake states (Guevara Erra et al., 2016; Mateos et al., 2017). Whereas in some studies connectivity was analyzed using phase synchrony of electrophysiological recordings, any other method which allows for estimation of the number of connectivity configurations can be used, like correlation between signals of functional neuroimaging (Hudetz et al., 2014). The inverted U present in the figure has appeared before in this field, sometimes associated with the notion of more complexity at the top of the curve (Tononi et al., 1998), the global concept being that consciousness requires medium values of certain features of cell assemblies, e.g., not too high or low synchrony or correlations (Tkačik et al., 2015).

Please note that *proposing a maximization of the number of connectivity patterns does not at all mean randomness in brain activity*. To start with, there cannot be pure randomicity in neural tissue if only because the activity is constrained by anatomical (structural) connections and biophysical features (e.g., synaptic plasticity). The randomness that one may perceive is just our perception of the large number of possible configurations (microstates) during awake states, such that when an observation/measurement is made to determine what microstate is present we cannot anticipate it because of the very large number; however, during an epileptic seizure, with many fewer microstates, we would be less “surprised” to find a particular microstate. It is this “surprise” to which we attribute randomness. This perception, incidentally, underlies the common fallacy in which entropy is associated with randomness (Ottinger, 2005).

The inverted U as shown in the figure gives us the possibility to compute an index to determine the “optimality,” so to speak and in an intuitive sense, of brain function. We note that the IIT proposes as well an index of consciousness by a quantity Φ, although empirical assessment of this factor is challenging because it is unfeasible to measure except in simple model systems. In contrast, our index can be computed in any neurophysiologic recording montage because it offers a number of microstates relative to the number of sensors in the montage. It is basically the number of configurations of the correlations extracted by analytical methods from a number of signals. Since most correlations are determined pairwise, then the inverted U is simply the plot associated with the binomial coefficient of “*n* signals choose 2,” $\left(\begin{array}{c} n\\ 2 \end{array}\right)$, which can be in general $\left(\begin{array}{c} n\\ k \end{array}\right)$ if more than pairwise correlations are analyzed. This gives the total number *N* of possible pairs of signals given a specific channel montage. After an experiment, if *p* pairs of signals are found to be “connected,” then we apply again the binomial coefficient to estimate C, the number of possible combinations of those *p* pairs: C= $\left(\begin{array}{c} N\\ p \end{array}\right)$ = N!/p!(N-p)!

In sum, all these calculations represent the relatively simple combinatorial problem that given a maximum total of *N* pairs of connected signals, in how many ways our experimental observation of *p* connected pairs can be arranged. The only problem arises when the montage provides many signals such that the estimation of C is not feasible. But this is very easy to solve, as one can take the logarithm of C and then use the well-known Stirling approximation for large n: log(n!) = nlog(n) – n (the Stirling approximation is frequently used in statistical mechanics). Using this approximation, and after some basic algebra, one finds the following formula that approximates the number C of connectivity configurations: ln(C) = N ln (N/N-p) - p ln (p/ N-p). And even simpler, we also note that for the coefficient $\left(\begin{array}{c} n\\ 2 \end{array}\right)$ the maximum is attained at $\left(\begin{array}{c} n\\ 2 \end{array}\right)/2$, hence it may be just enough to compute not C but only *p* (pairs of signals connected) and examine the distance (difference) from that maximum, *n* being the number of signals. In our efforts to simplify, this could be our crowning achievement.

In some studies (Guevara Erra et al., 2016; Mateos et al., 2017) the logarithm of the number of microstates was taken to represent the (Shannon or Gibbs) entropy of the system, but this entails the assumption that all neural connections are equiprobable, which is far from reality. There is no real need to assume that ln(C) represents any particular entropy, as opposed to a simple index of connectivity configurations that provides an idea of the distance of this number to the maximal of the system; the closer it is, the better the brain is processing the information. We prefer not to use terms like “more conscious” or “less conscious” because, as explained in section 2, we opt to define consciousness by its features, and also because we consider it analogous to cognition, all of which renders translation of levels of consciousness/cognition to single indices rather difficult.

### 5.2 Proposed empirical tests of the MaxCon framework

There are feasible experimental tests of our hypothesis, the obvious one being to directly measure the configurations of connectivity patterns. Only a few studies have done this (discussed in section 6). Although it is currently impossible to trace all brain cell chains contributing to a behavioral/cognitive action, the main option is to study correlations of activity. The expectation from our proposed theory is that unconscious states will have fewer configurations of connections among brain areas. We will see in the next section that this has already been indicated by many studies.

Recalling our idea of defining consciousness through an enumeration of its features, one could propose that conscious moments containing important sensory deprivation will lead to fewer configurations of connections. This has been reported in subjects with their eyes closed (Guevara Erra et al., 2016). Without visual input an important part of sensing the environment is removed –in keeping with the prominence of visual processing in humans, interrupting visual inputs to the brain results in considerable changes in its dynamics (the appearance of prominent alpha waves)– so an individual might be labeled as “less conscious” without visual information; having the eyes closed may not change the global conscious state much, but it reduces the optimality of sensorimotor processing. Therefore, *our framework incorporates as well not only states of global consciousness but also the optimality of sensory processing* (we use the term “optimal” in the intuitive sense of neural processing conferring on an organism greater adaptability). This is reasonable because, as David Marr advised, to understand a neurobiological structure one has to understand what it is the structure is attempting, as a whole, to achieve: “if one hopes to achieve a full understanding of a system as complicated as a nervous system […] one must be prepared to contemplate different explanations at different levels of description that are linked, at least in principle, into a cohesive whole even if linking the levels in complete detail is impractical” (Marr, 1982).

Going beyond empirical tests, there is the possibility to formulate predictions of the theory with a formula including a function of the probability of connections and the inputs from the surroundings. In this sense, theoretical frameworks grounded in the intrinsic dynamics of a system coupled to an environment using some sort of viability functions have been advanced (Kolchinsky and Wolpert, 2018). It is important to include the environment because our behavioral responses are not fixed but driven instead by an environment, a context, and so as cognition is grounded in sensorimotor coupling, consciousness cannot be separated from function (Cohen and Dennett, 2011; Engel et al., 2013). We are presently working on this more theoretical aspect.

### 5.3 On the location of consciousness

With these ideas we can try to answer common questions that appear in the field related to the issue of locating consciousness. For example, why is the cerebellum thought not to be necessary for consciousness (Yaron et al., 2022)? We saw before that some theories propose specific main locations, normally disagreeing on the contribution of cortical regions and omitting subcortical influences. Attention to many neurobiological details brings about the peril of losing the perspective that sees an organism as a functioning whole. Having in mind the bidirectional flow of information, the aforementioned neural closure, where cortical nets are reciprocally connected (Felleman and van Essen, 1991), along with the teachings from split brain patients, subliminal perception, etc., it is difficult to posit a localized region specific for conscious awareness. Neural mechanisms have been proposed for subliminal, preconscious and conscious processing (Dehaene et al., 2006), making it possible that diverse configurations of connections process unconscious transformations contributing to task performance (an element of cognition) but not to awareness (Lau and Rosenthal, 2011). Considering all of the many features of consciousness, it is unfeasible to identify a regionally localized area because those features are processed in various combinations of neural networks. As such, we consider that *those neural connectivity configurations throughout the nervous system are, themselves, consciousness/cognition*, each configuration representing some aspect of the sensorimotor transformations the individual is undergoing. This coidentification of consciousness and cognition with the multiplicity of network configurations in the brain is similar to aspects of the IIT, without recourse to temporal–parietal-occipital localization or the Φ factor.

Indeed, even decorticated humans may be conscious, and of course the brainstem is fundamental for consciousness. It all depends on the function, which is in part derived from the connectivity among different brain areas. So our answer as to why the cerebellum is not needed for consciousness is that the cerebellum is indeed needed for some aspects of consciousness but, due to the special connectivity among cerebellar regions and with other brain areas (it has afferent and efferent connections with cerebral cortex), it is not necessary for higher features of consciousness like self-awareness. The cerebellum has a relatively constant structural configuration, and while there is recurrence in cerebellar circuits, this deviates from the strict closed loop reciprocality seen in cortical networks (Houck and Person, 2014; Benagiano et al., 2018). Therefore, we speculate that the cerebellum does not display the multiplicity of configurations of neural connections that cortical lobes in the cerebral hemispheres have, perhaps because of the special arrangements of structural cerebellar circuitries that, with less widespread re-entry, do not allow for conscious, top-down prediction or meta-processing of information.

To sum up, to assign an essential role to a brain area in consciousness would be like attributing to a specific neurotransmitter a major role in this phenomenon. And in fact acetylcholine has been considered a “neurotransmitter correlate of consciousness” on account of its actions in attention and arousal (Perry et al., 1999), however, one could say the same thing about glutamate or histamine. Nonetheless, one can assign a particular feature of consciousness, –e.g., attention– to a particular region –e.g., the frontal lobe–, but even then it is mostly a variety of interconnected cortical and subcortical brain regions that are involved.

### 5.4 On the mystery of consciousness

The source of the apparent “mystery” of consciousness lies in the fact that consciousness is a subjective experience. As aforesaid, in the final analysis all consciousness is consciousness (awareness) of something, or, correspondingly in Antonio Damasio’s words, consciousness is the feeling of what happens (Damasio, 1999). This apparently self-evident claim is of essential significance because this simple fact points to a most basic aspect of consciousness: perception, and how our brains interpret those percepts. We can make it as complicated as we want talking about access or phenomenal consciousness, or subliminal processing, etc., but in the end it all starts with perception. The body-subject perspective of Maurice Merleau-Ponty may be the only practical viewpoint from which to begin to understand consciousness (Merleau-Ponty, 1945). Perception needs segregation to process sensorimotor inputs in localized brain regions but also integration of those localized “computations” (so to speak) to perceive the whole. And for this a wide array of configurations of neural connections is needed because nervous systems are continuously bombarded with inputs from both the environment and the body. This is in a nutshell the gist of our theory.

Is then our framework just a neural correlate of consciousness? There is intense research on the neural correlates of consciousness, and with good success as some features of consciousness have been associated with certain brain regions. In the final analysis, as Henri Poincaré told us, relations are all we can study: “the aim of science is not things themselves […] but the relations between things; outside those relations there is no reality knowable” (Poincaré, 1905). From these relations, or correlations, neural theories are derived, and where our theories border with the considered “unknown” (subjective awareness, qualia…) constitutes philosophical provocation, motivating attempts to explain the material mind by going beyond it, to dualism or deeper mysticism. That the conscious experience processed by our brain cannot be reduced to neural activity is an assumption many scholars seem to easily accept –the dualistic dichotomy, the everlasting mind-matter distinction. Others, though, do not see such profound mystery, proposing, e.g., that “a systematic experimental project could help move beyond the hard problem” (Klein and Barron, 2020), or stating “It looks like scientists and philosophers might have made consciousness far more mysterious than it needs to be” (Seth, 2016), and some have attempted to demystify consciousness (Feinberg and Mallatt, 2018; chapter 7 in Perez Velazquez and Nenadovic, 2021). The idea that a theory of consciousness must account for processes underlying introspection, or the experiential (phenomenal, subjective) dimensions of consciousness, led to another dichotomy, with scholars debating between access consciousness and phenomenal consciousness. The latter is related to the “hard problem,” about which a lot has been written. We will just mention that the notion of phenomenal consciousness, at least according to some, is the raw experience (of or about something) considered independently of any impact on behavior, and these introspective experiences are what are called qualia. Considering our words above that consciousness/cognition cannot be separated from behavior, nor from an individual brain’s embodiment in an environment, a position long held by others (Lashley, 1923a,b; Merleau-Ponty, 1945; Cohen and Dennett, 2011), we do not see any particular advantage in thinking about qualia in this manner.

In any event, it is known that brain regions contain representations of the body and of its environmental circumstance, whereby sensations we call introspection, interoception or proprioception emerge. Sensations are the speciality of the nervous system. Whether coming from a reflex arc or more complex processing, sensations are what nervous systems fabricate; and these fabrications depend on the neural circuitry. As an illustration, what does a bird singing evoke? In a human, several emotions/feelings; in a hungry predator, a move toward hunting the bird. The special thing about what the songbird evokes is determined by the structure, the arrangement of the neural circuitries that store information, memory, in the different creatures. We may be mistaken in attributing to sensation a nonphysical character, because this is just what the (neuro)physics does: sense and react. The nervous system evolved to sense, to produce sensations, and it is but a matter of degree the difference in sensation between a reflex arc and more sophisticated “arcs” in our brains. According to our view proposed in this paper, the more possible interacting configurations of brain areas exist, the more intricate and elaborated the sensation is.

### 5.5 Practical applications of MaxCon: arousing dormant brains

Our framework may have practical applications in the clinic. If few configurations of connections is problematic for healthy cognition, then why not try to increase the connectivity patterns? There seems to be chronic underactivation of brain circuits in patients with severe disorders of consciousness –coma, vegetative state, minimally conscious state– but these circuits are potentially recruitable into large-scale networks by neurostimulation. Evidence for this notion can be found in the well-known study of Schiff et al. (2007) where electrical deep brain stimulation (DBS) was applied to the intralaminar thalamic nuclei to try to aid the residual brain activity that was present in a patient who had remained in a minimally conscious state for 6 years. The stimulation caused an overall increase in cortical excitability because those subcortical nuclei project to almost all cortical areas. As a result, the patient partially recovered (more comments in section 6). We note that there are also noninvasive neurostimulation methods such as transcranial direct current stimulation or transcranial magnetic stimulation, although depending on the location of the desired target inside the skull invasive methods may be more appropriate than extracranial techniques.

We offer the following specific approach based on the study of fluctuations in neural network connectivity. Investigating brain patterns in conscious and unconscious patients, it was found that some neural patterns of a fully awake state appear, albeit transiently, in the vegetative or coma states (Demertzi et al., 2019). This observation suggests that there are brief moments during which the brains of such patients are trying to become active, so to speak, and this activity will be translated into more fluctuations in the patterns of synchrony. Hence, a conceivable approach to a closed-loop DBS (or maybe noninvasive neurostimulation) protocol to “awaken” these patients would be to analyze in real time neural synchrony using, e.g., electroencephalography (EEG), and when some important fluctuations are found, this would be a signal that a partial recovery may be about to occur and the moment when neurostimulation could be applied to sustain and enhance that residual activation, recruiting long-range neural connections. Therefore, while not too many studies have been done, the available evidence indicates that a precisely timed neurostimulation could help dormant brain circuits become more active and thus relieve the unresponsiveness of the patients.

## 6 Empirical support for the tendency to maximize configurations in which neural networks can communicate as a fundamental feature of conscious awareness

We will now examine empirical results in line with predictions supporting our proposal. We should note the criticism about a lack of meaningful empirical tests of theories of consciousness (Fleming et al., 2023), but if carefully assessed, putting together the common thread of many reports, the results hint at the subject matter that we are discussing here. Indeed, a quick survey of the literature reveals that consciousness/cognition research makes use of the (many) concepts of complexity with a variety of complexity-related measures, and a trend of converging evidence is becoming apparent, in that the complexity of neural activity decreases during loss of consciousness (recently reviewed in Sarasso et al., 2021). This evidence goes along with the view of consciousness as an *integrated dynamic process capable of achieving a large number of configurations of neural network connections*: the more ways in which the information can be exchanged among cell ensembles, the better for awareness and brain health.

One immediate prediction, as mentioned in the previous section, considering the proposal that larger numbers of configurations of connections represent an optimal brain “web” to navigate the environment, resulting in greater adaptability, is that animals in so-called enriched environments should present larger numbers of configurations of connections in their nervous systems. It has been known for a long time that among rodents raised from weaning in an enriched environment, various brain alterations are observed including increased cortical thickness, more dendritic spines, more synaptic contacts and extensive branching (Diamond et al., 1964; Nilsson et al., 1999). While these observations suggest that there may be increased numbers in the configurations of connectivity in enriched environments, these are not strict demonstrations because, to our knowledge, no study has computed the number of connectivity configurations in these conditions. Nevertheless, the reports that the functional modifications due to these environments include increased high-dimensional coding and functional connectivity “that might enhance pattern separation by providing larger activity space […] to embed different representations with minimum overlap” (Emery et al., 2022) are a more direct hint supporting our idea.

A very direct support for our framework is the evidence for the tendency to maximize configurations of neuronal connections in conscious states derived from phase synchrony studies. In brief, studies using invasive and noninvasive electrophysiological brain recordings in different states of consciousness (wakefulness, sleep, seizures and coma) evaluating the phase synchrony between pairs of signals found that the number of configurations of (pairwise) connectivity –the microstates aforementioned– associated with conscious awareness is larger than the number during unconscious states (Guevara Erra et al., 2016; Mateos et al., 2017). Using the index shown in section 5.1, conscious states were closer to the top of the inverted U (Figure 2). Whereas no link was established with the psychological level of mental states, it is conceivable that the global “mental macrostate” during normal wakefulness corresponds to the combination of all that sensorimotor processing carried out by the different microstates, hence the number of microstates should be larger than that of the macrostates associated with unconscious states. These results are in parallel to another study using resting state EEG that found increased spatial and temporal variability of brain networks to be associated with the level of consciousness (Cai et al., 2020).

There are other studies using neuroimaging data reporting a more constrained repertoire of neurodynamic states during unconsciousness (reviewed in Mashour and Hudetz, 2018). As a representative example, a functional magnetic resonance imaging (fMRI) study reported that brains of unresponsive patients in vegetative or minimally conscious states had smaller probabilities to transition between neural activity patterns, with the authors concluding that “consciousness rests on the brain’s ability to sustain rich brain dynamics” (Demertzi et al., 2019); similar observations were made in pathological and pharmacological low-level states of consciousness, including reduced network interactions and more homogeneous and constrained neural dynamics (López González et al., 2021); and in the fewer transitions in “between-network” connectivity patterns during general anesthesia (Golkowski et al., 2019).

In addition, there is a multitude of studies reporting results suggesting that conscious awareness needs a large number of configurations of neural network connections, and we say “suggesting” because the analytical methods used in these studies do not strictly determine the number of connectivity configurations. For instance, a study based on scalp EEG data using phase synchrony and graph theory-based analyses reported decreases in the communication between distant brain networks and increases in the local connections in patients with disorders of consciousness (Rizkallah et al., 2019). Hudetz et al. (2014) graphed a dispersion index versus an activation level (their Figure 7B) and proposed that consciousness resides at the top of the curve: this ‘activation level’ could correspond to the number of signals/networks that are “connected” whereas their ‘dispersion index’ could be interpreted as the number of cell connection combinations. Then, Afrasiabi et al. (2021) used the entropy of local field potentials and quantified the uncertainty about the system state, which reflects the number of available states, with higher entropy indicating more uncertainty and thus more possible system configurations, “which is a putative feature of consciousness” in their words. Indeed many studies have used various entropies applied to neurophysiological time series (Keshmiri, 2020). We offer here only a brief comment to point out that two of those entropies many times used, sample entropy and permutation entropy, in the end reflect the variability in neural network connections because these entropies measure the predictability of time series recordings, therefore the manifestation of the fluctuating collective activity of many brain cells.

In closing, many studies reporting greater complexity of brain dynamics during conscious awareness can be interpreted within the MaxCon framework as a larger number of connectivity configurations. This is also indicated by examinations of cognitive tasks that require reporting stimuli, such as a study recording multiunit activity in monkeys that demonstrated that the reported stimuli were associated with strong sustained activity in the frontal cortex and in several other areas like temporal and parietal cortices (Van Vugt et al., 2018); these very widespread activations can be inferred to be sustained by greater ways of connectivity among brain areas.

Similar indications that healthy cognition and conscious awareness need a substantial variability in neural connectivity are offered by studies of pathological cases. One that is of central importance because it suggests a means to “awaken” the pathologically dormant brain –mentioned in section 5.5 when discussing practical applications of our framework– is the study where some residual activity was found in restricted brain regions in a patient who was in a minimally conscious state and clinicians applied DBS in an attempt to augment this little activity so that it might become larger and enable long-range neural activations. Because the DBS electrodes were implanted in the intralaminar nuclei of the thalamus, this placement favored that the net effect of the stimulation would be an overall increase in excitability of the cortex (since the intralaminar nuclei project to almost all brain cortical areas); one could even say that the stimulation enhanced the “internal brain noise.” As a result, some cognitive behaviors and functional limb control increased during periods in which DBS was on as compared with periods in which it was off –the patient thus partially recovered (Schiff et al., 2007). It is then conceivable that upon thalamic stimulation, some of the many cortical configurations of neural connections that the patient’s brain had maintained while healthy were re-activated. In traumatic brain injury, studies have revealed that the temporal and spatial variability of phase synchronization among EEG signals increased as patients emerged from coma, and this variability correlated with clinical outcome (Nenadovic et al., 2008, 2014). Similarly, during generalized seizures when patients become unresponsive, there is lower spatial and temporal variability of neural synchrony (Chavez et al., 2005; Garcia Domínguez et al., 2008; Perez Velazquez et al., 2019). All these results in pathological cases indicate that fewer combinations of connections among brain areas occur during unconsciousness.

Finally, let us mention that computational and theoretical studies endorse the main message of our proposal, indicating that the variability in the patterns of brain activity arising from the maximization of fluctuations in synchrony is fundamental for a healthy brain (Garrett et al., 2013; Vuksanovic and Hovel, 2015).

Summing up, there seems to be experimental and neurocomputational evidence supporting our proposal for a sufficient condition for consciousness to emerge, namely the multiplicity of configurations in which neural networks can communicate, or exchange information. In simple words: the more ways brain networks can communicate, the more “aware” an entity is.

## 7 Concluding remarks

We have tried to offer a very concrete, neurophysiologically framed theory based on the maximization of numbers of brain network connections, rather than elaborating on notions like complexity, entropy or information, with their overlapping interpretations. Although it is true that maximization of the number of connectivity patterns can be captured by some of these measures, there will always exist a certain arbitrariness as to what method of computation and what notion is used, as elaborated in the Supplementary material. Our proposal, from the level of description of the interactions/relations among cell networks, *underscores the tendency to maximize the number of configurations of brain network connections* needed for appropriate, adaptive brain information processing –constrained by neuroanatomy (structural connectivity), biophysical features and the environment–, and the main mechanism governing the emergence of neural dynamic patterns: the fluctuations in cellular collective activity. Unlike other theories, ours proposes a very clear neurobiological mechanism –the number of connectivity configurations, not confined to one or a few specific areas– that can be computed with diverse recording and analytical methodologies.

## Data availability statement

The original contributions presented in the study are included in the article/Supplementary material, further inquiries can be directed to the corresponding author.

## Author contributions

JP: Writing – original draft, Writing – review & editing. DM: Writing – review & editing. RG: Writing – review & editing. RW: Writing – review & editing.
