# Supplementary material for: Unifying biophysical consciousness theories with MaxCon: maximizing configurations of brain connectivity
Source: Front Syst Neurosci. 2024 Jul 29;18:1426986. doi: 10.3389/fnsys.2024.1426986 (PMC11317472; doi:10.3389/fnsys.2024.1426986)
Supplement: Supplementary file 1 [file Appendix.PDF]

<u>Supplementary Material to: *Unifying biophysical ToCs with MaxCon*, by Perez Velazquez et al.</u>

APPENDIX: Technical arguments about our groundwork and fundamental perspective

With regards to our approach to defining consciousness by the enumeration of features, we must consider that basic concepts in science, e.g., linearity/nonlinearity, are defined in terms of enumerating features, hence it should not be unconceivable to define consciousness (and cognition, and life too) using this method. Defining consciousness in this manner solves two basic problems that have been persistently discussed: "Is it possible to study consciousness scientifically?" and "Which organisms are conscious?" The answer to the first query is explained in section 2, and the answer to the latter is that all organisms have some features of consciousness, even microorganisms and plants, to the extent that they sense and react to the environment, so in this wider definition all entities are "conscious". Note that this is not exactly the common panpsychism notion, at least in the sense of panexperientialism, because the view is not that fully conscious experience is ubiquitous: paramecia may sense and react to environmental factors but (most likely) they are not experiencing these responses as other more advanced animals do; the sense of agency needs some self-awareness that only sophisticated brains can generate.

Regarding "neural connectivity", whereas many studies evaluate this in several ways, in reality the analyses reveal only *correlations in activity*; for instance, in the case of phase synchrony it is a correlation between the phases of oscillations. We will not discuss the issue of using as synonymous the notions of synchrony and connectivity, and refer the reader to many articles where several types of connectivity have been described. We just note the unfeasibility of an accurate, realistic estimation of "real" functional connectivity, which would necessitate individual cell recordings from entire cell ensembles as well as knowledge of structural connectivity details; hence we use the accepted version under the assumption that the (co)relations of activities may represent, at minimum, some aspect of a functional connectivity. In any case, there is basically no choice: simplification is needed because the state space of neural dynamics if individual cells are considered is enormous, therefore studying correlations of activity helps to reduce the dimensionality of that state space. There are many empirical observations about the association of changes in neural correlated activity (which normally is evaluated as synchrony) and behaviours; support for the notion that coordinated activity in the brain is the biophysical underpinning of behavioural outcomes can be found in several studies (e.g., Niessing and Friedrich, 2010).

We are proposing the notion of microstates as neural connectivity configurations (Figure 1), but will not delve into the discussion of whether these configurations and thought processes are representations of the same thing, although this is "a reasonable working hypothesis" (Little, 1974), reflecting perhaps just different levels of description. As well, we note that the word 'information' appears in the text but we do not go into discussing what information is and what type of information processing applies to nervous systems —a topic that has been treated in many studies, e.g., Piccinini and Scarantino (2011) or Timme and Lapish (2018)— rather we use the term in its intuitive meaning.

We focus our approach on the interactions among cell networks/brain areas. In a general sense, interactions determine the progression of natural phenomena (Perez Velazquez, 2009). Thus, our main feature is the interactions of networks of cells that bring about the emergence of patterns of organised activity which determine behaviours (Wright and Liley, 1996; Nunez, 2000; Hudetz et al., 2014). The perspective of interactions/relations among cell networks helps to make sense of neuroscientific data. We note that we are not dismissing the microscale of cellular activity, because after all the meso/macroscale is determined by the micro level, but one has to choose to focus on some level to make sense of very complex dynamics. How structural and functional connectivity shape neural dynamics is described in many texts (e.g., Sporns, 2022; de Schotten & Forkel, 2022) so we will not review it here.

The principle of organization of neural dynamics that we offer, expounded in section 3, about the tendency to maximise the number of configurations of connections among cell networks, is plausible considering some major aspects of nervous system activity and function. First, it reflects the variability of neural dynamics, which provide a diversity of states for different patterns of activity needed for the appropriate sensorimotor transformations to promote survival of the organism. In fact, it is currently accepted that fluctuations of spontaneous brain activity represent a fundamental principle of brain (dynamic) organization, even in unconscious states (Pinsk and Kastner, 2007). On the whole, fluctuations of macroscopic variables that account for microscopic processes are fundamental to understand phase transitions —bifurcations in dynamical system terminology, which have been described in brain dynamics (Haken, 1998).

We need to draw attention to a confusing aspect that arises when trying to understand and compare theories or experimental observations. The problem we face in modern neuroscience (and perhaps in most of biology) is that the use of concepts, many from the fields of thermodynamics and statistical mechanics (e.g., equilibrium, entropy, organization, complexity, information), as analytical techniques, makes it difficult to compare the results derived from diverse studies using diverse methods for quantifying, say, complexity or entropy. As Thurner et al. (2017) have written about the notion of entropy (H): “The naive use of the expression H as a one-size-fits-all concept will inevitably lead to confusion and nonsense”. These concepts have several definitions, and a specific meaning only in specific circumstances. We are prone to making the mistake of not seeing the essence of the results, of not transcending, so to speak, what the application of those methods reveals in terms of the underlying neurophysiology. This concern over the variety of analytical methodologies and their application to multiple phenomena has been voiced by several scholars: “discrepancies will be found also in the case of complexity-related measures […] this is to be expected if only one considers the number of different methods and spatiotemporal scales at which complexity is estimated […] it will be critical for future studies to precisely identify how they define and quantify complexity” (Sarasso et al., 2021). Let it be clear that we are not censuring the use of these concepts and methods, they are *very valuable in so far as they are used with a specific notion and in particular experimental situations*, but extreme care should be taken when invoking these notions/results in a global manner to describe complex phenomena.

It may be as well needed to indicate —for the punctilious reader— that frameworks and theories may not always be unambiguously separated. It is normally understood that frameworks are structures that support theories, while theories are formulated to try to understand, explain and predict phenomena. In our text we deal with frameworks and theories without making fundamental distinctions, as this gives us a wider perspective in our attempt to understand how consciousness and cognition arise from the organization of the nervous system.

References

de Schotten, M. T., & Forkel S. J. (2022). The emergent properties of the connected brain. *Science, 378,* 505-510. doi:10.1126/science.abq2591

Haken, H. (1998). *Information and Self-organization*. Berlin: Springer.

Hudetz, A. G., Humphries, C. J., & Binder, J. R. (2014). Spin-glass model predicts metastable brain states that diminish in anesthesia. *Frontiers in Systems Neuroscience, 8,* 234. doi:10.3389/fnsys.2014.00234

Little, W. A. (1974). The existence of persistent states in the brain. *Mathematical Biosciences, 19,* 101-120. doi:10.1016/0025-5564(74)90031-5

Niessing, J., & Friedrich, R.W. (2010). Olfactory pattern classification by discrete neuronal network states. *Nature, 465,* 47-52. doi:10.1038/nature08961

Nunez, P. L. (2000). Toward a quantitative description of large-scale neocortical dynamic function and EEG. *Behavioral and Brain Sciences, 23,* 371-437. doi:10.1017/S0140525X00003253

Perez Velazquez, J. L. (2009). Finding simplicity in complexity: general principles of biological and nonbiological organization. *Journal of Biological Physics, 35,* 209-221. doi:10.1007/s10867-009-9146-z

Piccinini, G., & Scarantino, A. (2011). Information processing, computation, and cognition. *Journal of Biological Physics, 37,* 1-38. doi:10.1007/s10867-010-9195-3

Pinsk, M. A., & Kastner, S. (2007). Neuroscience: unconscious networking. *Nature, 447,* 46-47. doi:10.1038/447046a

Sarasso, S., Casali, A. G., Casarotto, S., Rosanova, M., Sinigaglia, C., & Massimini, M. (2021). Consciousness and complexity: a consilience of evidence. *Neuroscience of Consciousness, 2021,* niab023. doi:10.1093/nc/niab023

Sporns, O. (2022). The complex brain: connectivity, dynamics, information. *Trends in Cognitive Sciences, 26,* 1066-1067. doi:10.1016/j.tics.2022.08.002

Thurner, S., Corominas-Murtra, B., & Hanel R. (2017). Three faces of entropy for complex systems: Information, thermodynamics, and the maximum entropy principle. *Physical Review E, 96,* 032124. doi:10.1103/PhysRevE.96.032124

Timme, N. M., & Lapish, C. (2018). A tutorial for information theory in neuroscience. *eNeuro, 5,* ENEURO.0052-18.2018. doi:10.1523/ENEURO.0052-18.2018

Wright, J. J., & Liley, D. T. J. (1996). Dynamics of the brain at global and microscopic scales: Neural networks and the EEG. *Behavioral and Brain Sciences, 19,* 285-295. doi:10.1017/S0140525X00042679
